# Recycling Endosomes and Viral Infection

**DOI:** 10.3390/v8030064

**Published:** 2016-03-08

**Authors:** Sílvia Vale-Costa, Maria João Amorim

**Affiliations:** Cell Biology of Viral Infection Lab, Instituto Gulbenkian de Ciência, Rua da Quinta Grande, 6, 2780-156 Oeiras, Portugal; svcosta@igc.gulbenkian.pt

**Keywords:** animal viruses, intracellular membranes, recycling endosome, Rab GTPases, Rab11, phosphoinositides

## Abstract

Many viruses exploit specific arms of the endomembrane system. The unique composition of each arm prompts the development of remarkably specific interactions between viruses and sub-organelles. This review focuses on the viral–host interactions occurring on the endocytic recycling compartment (ERC), and mediated by its regulatory Ras-related in brain (Rab) GTPase Rab11. This protein regulates trafficking from the ERC and the trans-Golgi network to the plasma membrane. Such transport comprises intricate networks of proteins/lipids operating sequentially from the membrane of origin up to the cell surface. Rab11 is also emerging as a critical factor in an increasing number of infections by major animal viruses, including pathogens that provoke human disease. Understanding the interplay between the ERC and viruses is a milestone in human health. Rab11 has been associated with several steps of the viral lifecycles by unclear processes that use sophisticated diversified host machinery. For this reason, we first explore the state-of-the-art on processes regulating membrane composition and trafficking. Subsequently, this review outlines viral interactions with the ERC, highlighting current knowledge on viral-host binding partners. Finally, using examples from the few mechanistic studies available we emphasize how ERC functions are adjusted during infection to remodel cytoskeleton dynamics, innate immunity and membrane composition.

## 1. Introduction

Host membranes are targeted by many viruses. Well-explored cases include the usage of membranes of the endoplasmic reticulum and of the Golgi by positive-stranded RNA and double-stranded DNA enveloped viruses ([1,2,3] for recent reviews). Advances in electron microscopy in the 1950s revealed prominent alterations in the architecture of infected cells, including extensive rearrangements of cellular membranes [4,5]. This prompted investigating the mechanisms sustaining viral-mediated membrane reshaping and the identification of their role as specialized sites for viral replication. Technological breakthroughs, including tomography and correlative light and electron microscopy, permitted identification of subtle changes, leading to the postulation that the usage of host cell membranes is a viral hallmark. Inspection of a wide range of viral-induced membrane alterations originated two interesting concepts. The first is the tremendous flexibility in viral strategies for targeting the same surfaces, and the second is the identification of viral developed programs to target membranes from most cellular organelles, namely the endocytic pathway, mitochondria and peroxisomes. These studies also provided evidence for additional roles these structures play, including modulating host antiviral responses, shaping lipid environment, and facilitating viral entry and exit from the cell [1,2,3,6,7,8,9,10]. The endocytic recycling compartment (ERC) was one of the compartments that recently emerged as being subverted during infection by an increasing number of pathogens with relevance to human health [11,12,13]. Initially, its function was believed to be restricted to transporting virion components, but this view is rapidly changing to accommodate additional roles that are explored below. Before progressing to describe in detail how viruses exploit the ERC, we will first discuss the machinery and principles governing trafficking to and from this compartment. This strategy will emphasize how little we know about viral targeting of the ERC despite the multitude of ways in which viruses could potentially alter their constitution and functions. The emerging picture, explored in this manuscript, is that viral interactions with the recycling endosome outcompete that of some cognate host factors, and this will impact their role. Given the large number of complexes identified at the healthy ERC, and the diversity in viruses and viral stages that use this system, it is plausible to assume that different strategies have been developed to orchestrate viral assaults. Their characterization, beyond the current state-of-the-art discussed here, will provide invaluable cues on the physiology of the healthy recycling endosome and the impact on human health.

## 2. Recycling Pathways Integrate the Endomembrane System

The survival of the compartmentalized eukaryotic cell depends on its ability to structure inter-organelle and inter-cellular communication systems that transport cargo from and to specific cellular locations, carrying updated information of the cellular status and extracellular environment, and machinery to respond to it. These signals are transported in vesicles that bud from donor organelles and fuse solely with specific acceptor compartments able to recognize and react to their composition, on account of specific networks of proteins and lipids [14,15,16].

**Figure 1:**
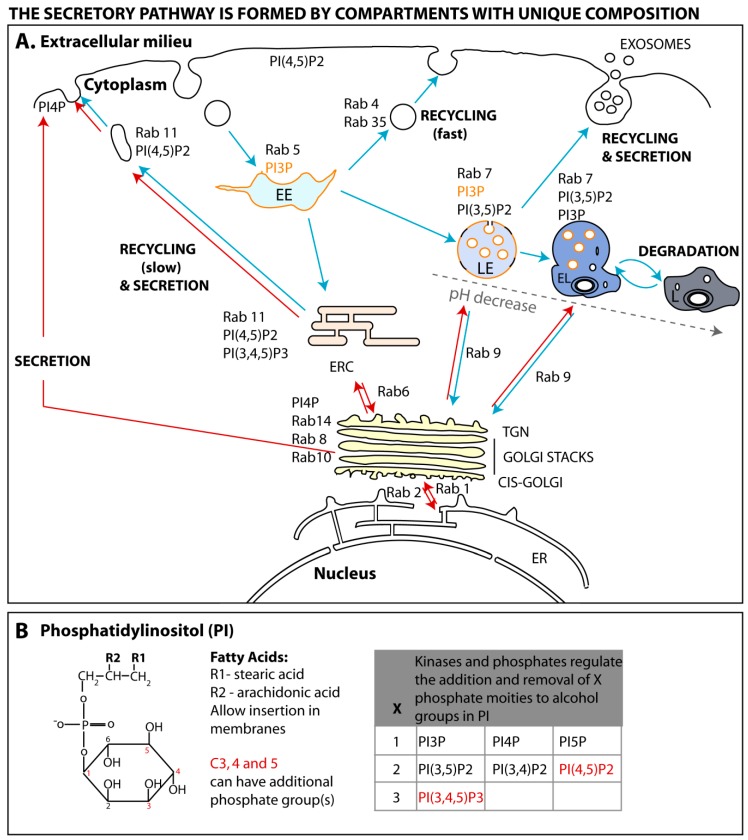
The endomembrane system of the eukaryotic cell and major Ras-related in brain (Rab) GTPases and phosphoinositides occupying its subdomains. (**A**) The eukaryotic cell is composed of several compartments. Trajectory of endocytosed material is depicted in blue arrows. Upon internalization, cargo is transported to the early endosome (EE), and either degraded upon passage through late endosomes (LE), endolysosomes (EL) and lysosomes (L), or recycled via a fast or a slow pathway that requires transfer from EE to endocytic recycling compartment (ERC). Secretory pathways from the trans-Golgi network (TGN) to the plasma membrane are highlighted in red. Note that at some points, exocytic and endocytic pathways intertwine. Rab GTPases and phosphoinositides occupy specific subcellular locations and are marked adjacent to corresponding compartments. Phosphatidylinositol-3-phosphate (PI3P) is highlighted in orange to facilitate illustration of its selective inclusion in internal luminal vesicles of LE; (**B**) The chemical structure of phosphatidylinositol is shown, highlighting carbon number of the sugar moiety in red. Fatty acids (R1, R2) in the glycerol moiety allow ligation to membranes. Carbon 3, 4 and 5 can be additionally phosphorylated by esterification of hydroxyl groups to originate seven isoforms, shown in the table. Isoforms found in the ERC (despite in minor amounts) are highlighted in red. Abbreviations include: PI4P: phosphatidylinositol-4-phosphate; PIP5: phosphatidylinositol-5-phosphate; PI3,5P2: phosphatidylinositol-3,5-biphosphate; PI3,4P2: phosphatidylinositol-3,4-biphosphate; PI4,5P2: phosphatidylinositol-4,5-biphosphate; PI3,4,5P3: phosphatidylinositol-3,4,5- triphosphate.

Cells internalize extracellular signals by endocytosis, a process comprising multiple pathways and complexes, whereby the plasma membrane is rearranged in a variety of ways to engulf exogenous macromolecules (reviewed in [17,18]). Regardless of the endocytic pathway, membrane rearrangements lead to the formation of vesicles of variable sizes, containing cargo, that detach from the surface and move into the cell. Internalized vesicles are delivered to the early endosome (EE), where material is sorted to several destinations (shown in Figure 1A and represented by blue arrows) [19,20]. Cargo can be sent for degradation through segregation to late endosomes that mature into lysosomes, recycled back to the surface by a fast or a slow process [21] or integrated in the secretory pathway upon retrograde transport to the trans-Golgi network (TGN). Cells send signals to the exterior using the secretory or exocytic pathway (represented in Figure 1A by red arrows). This system ensures that proteins and lipids are synthesized, modified, folded, incorporated into vesicles and transported to the plasma membrane, where material can be secreted or become resident at the plasma membrane upon vesicular fusion. There are constitutive and regulated secretory systems, the latter activated upon specific stimuli. Secretory and endocytic pathways share common interfaces. For instance, several routes originating at the TGN transport material to early and late endosomes, lysosomes, and ERC [22,23,24].

As stated above, this review focuses on the viral–host interactions occurring at the ERC, a system that ensures the recycling of some endocytosed material and the delivery of specific TGN material to the plasma membrane [20,21,25]. As a consequence, it regulates the composition of the cell surface, playing key roles in diverse crucial processes such as the establishment of cell–cell contacts [26], cell polarity and migration [27,28], nutrient uptake [29], cytokinesis [30,31], synaptic plasticity [32,33], immune response [34] and infection. With an increasing number of identified viruses exploiting the ERC, it is becoming crucial to characterize the mechanisms by which this compartment is being targeted and ascribe its role(s) in infection. One feasible approach is to compare the physiology of the ERC in healthy and infected cells. There has been much research on how endocytosed material is transferred from the EE to the ERC, to a lesser extent from the TGN to the ERC and from the latter to the plasma membrane.

## 3. The Endomembrane System Is Formed by Compartments of Unique Composition

In physiological conditions, each compartment is defined by a set of constitutive proteins, lipids and cargo. Their homeostasis is maintained by highly dynamic and reversible biochemical processes that occur at their surface to form outgoing vesicles, while also being docking sites for incoming vesicles. Directionality of transport is imprinted by the composition of protein scaffolds assembled on lipid membranes during biogenesis, and sequential recruitment/removal of factors promoting vesicular progression [12,35,36,37,38]. We will discuss how these scaffolds assemble and are regulated, focusing on two major classes of coordinators in vesicular trafficking: Ras-related in brain (Rab) GTPases and phosphoinositides.

### 3.1. Rab GTPases

The first proteins to be described to define a membrane subdomain belong to a large family of highly conserved GTPase proteins, called Ras-related in brain (Rab) proteins [39,40], whose activity depends on GDP/GTP association [41]. In simplified terms, GTP binding to Rabs allows recruitment of effectors that mediate vesicular movement on cytoskeleton and fusion with membranes. There are over 60 different Rabs encoded in the human genome [42], occupying specific subcellular localizations as illustrated in Figure 1A and reviewed in [37]. The activity of Rabs is not only spatially regulated but is also under functional and temporal control to ensure the reversibility and bi-directionality of the processes they govern.

#### 3.1.1. Rab GTPase Spatial Regulation

**Figure 2:**
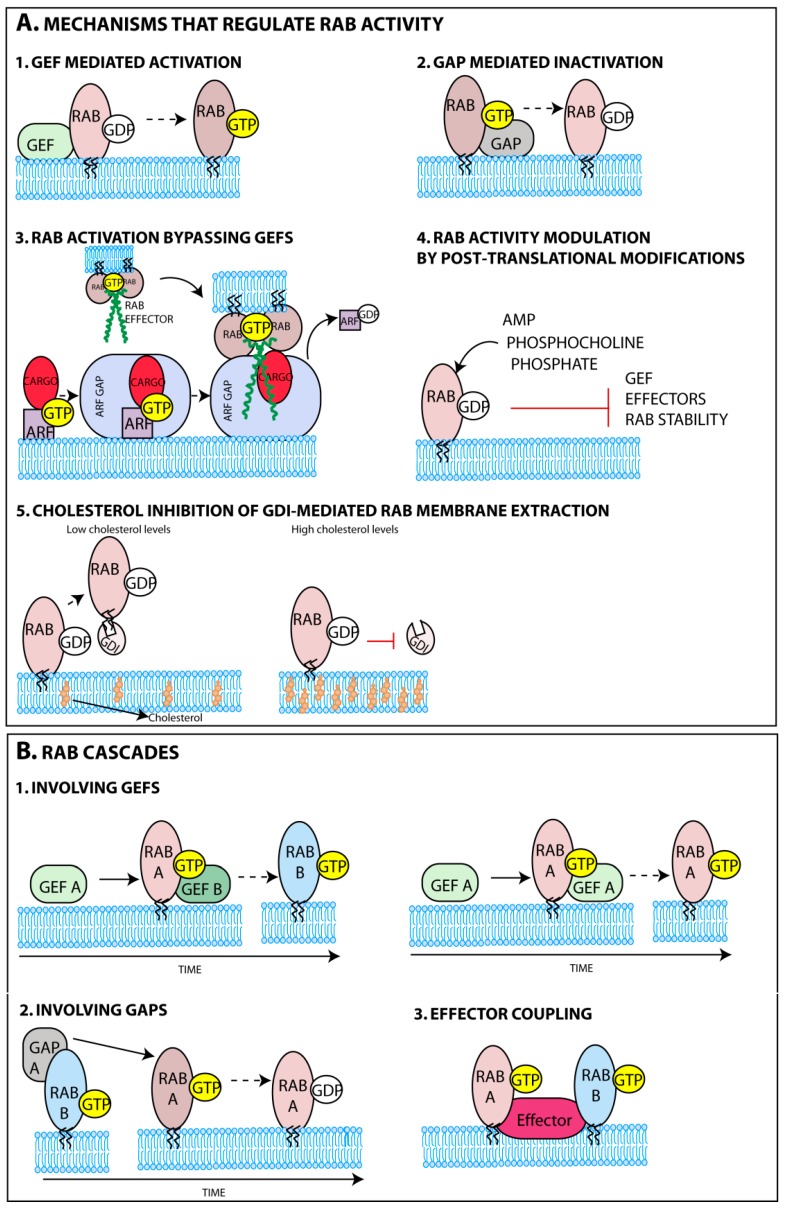
Rab GTPase spatial and temporal regulation. (**A**) Activation of Rab GTPases occurs through the exchange of GDP by GTP, a process catalyzed by guanine nucleotide exchange factors (GEFs), with a concomitant conformational change (1). Active Rabs attract multiple effectors and can be converted to the inactive state through GTP hydrolysis (2), which is accelerated by GTPase-activating proteins (GAPs). In certain circumstances, the recruitment of Rabs to membranes can bypass activation by Rab-GEFs. Activated ADP ribosylation factor (ARF) binds its cargo, and the dimer recruits the ARF GAP. The newly formed trimer has also affinity for an effector of a Rab, establishing a crosstalk between a ARF and a Rab (3). Alternatively, post-translational modifications can also regulate Rab function (4). High cholesterol content in membranes can reduce Rab extraction from membranes by inhibiting the activity of the corresponding guanine nucleotide dissociation inhibitory proteins (GDI) (5); (**B**) Rab GTPase conversion cascades are flexible mechanisms that allow crosstalk between Rabs. The activation (1) or inactivation (2) of a particular Rab can be made by a GEF or GAP, respectively, that was recruited by another Rab that acts upstream. The same Rab can also recruit its own GEF to maintain the activation state by positive feedback loop (1). Distinct Rabs can bind the same effector (3), a process called effector coupling.

**Figure 3:**
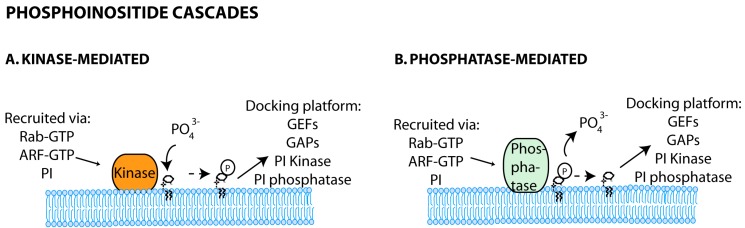
Phosphoinositide cascades. The phosphoinositide-mediated recruitment of kinases (**A**) or phosphatases (**B**) to act upon other phosphoinositides can initiate the formation of protein scaffolds, which regulate downstream membrane trafficking events.

The delivery of Rabs to membranes is facilitated by the post-translational addition of two prenyl groups to two cysteine residues of its C-terminal domain. This reaction is catalyzed by the enzyme geranylgeranyltransferase (GGT) with the help of a Rab escort protein (REP) [43,44,45]. While in the cytoplasm prenylated Rabs are found attached to guanine nucleotide dissociation inhibitory proteins (GDI) [46,47] and their targeting to membranes might be, in some cases, aided by GDI displacement factors (GDF) [48]. The major determinants for Rab targeting to membranes were shown to be guanine nucleotide exchange factors (GEFs) either alone or in conjunction with phosphoinositides or protein co-factors such as ubiquitin (Figure 2A-1) [49,50,51]. Elegant studies demonstrated that GEFs mistargeted to the mitochondria, by fusing a mitochondrial targeting sequence, successfully attracted their cognate Rabs. The analysis included well-established Rab-GEF pairs such as Rab5A-Rabex-5, Rab1A-DrrA, Rab8-Rabin8 [52], and Rab32/Rab38-BLOC3 [53]. GEFs’ affinity to a restricted number of Rabs and strategic positioning thus greatly contributes to the composition and functional compartmentalization of sub-organelles. The mechanisms governing GEFs occupancy at distinct membranes have been explained elsewhere [54,55]. In some cases, the recruitment of Rabs to a specific membrane was shown to bypass activation by Rab-GEFs (Figure 2A-3). This is illustrated in the *Drosophila* eye, for the transport of the cargo rhodopsin to specialized cilia, a process mediated by a complex network of proteins. Rhodopsin binds GTP-ARF4, an ADP ribosylation factor (ARF), in the Golgi [56]. ARFs that are regulators of vesicular biogenesis are also GTPases suffering rounds of activation and inactivation by ARF GEFs and GTPase-activating proteins (GAPs), respectively [57]. The ARF4 GAP, ASAP1, binds the heterodimer (ARF4, rhodopsin) [58] and recruits the family-interacting protein 3 (FIP3), an effector of Rab11a, hence recruiting activated Rab11a to the complex (Figure 2A-3) [59], while also switching off ARF4 [60].

From what has been said, there are many processes regulating Rab distribution and localization; all are prone to exploration during viral infection to putatively tailor membrane function in many different ways. Viral interference with these systems has not received a lot of attention, but recent evidence suggests that Rab11 membrane recruitment during influenza A virus (IAV) infection increases, by a mechanism that is still unclear, and this is crucial for efficient viral replication [61].

#### 3.1.2. Rab GTPase Temporal Regulation

Besides controlling subcellular localization of Rabs, GEFs also work as molecular switches (Figure 2A-1), catalysing GDP-to-GTP exchange that induces a conformational change and regulates the onset of Rab function [62,63]. The conformational change imposed by GTP binding, allows recruitment of a complex network of effectors, attracted sequentially, that mediate movement of vesicles, identification and fusion with acceptor membranes [64,65,66,67,68,69]. Once fusion with acceptor membranes is completed, guanine-activating proteins (GAP) accelerate GTP hydrolysis on Rabs (Figure 2A-2), reducing their affinity for effectors and terminating their activity [70,71].

In the GDP state, Rabs regain affinity for GDIs, are extracted from membranes and recycle to the initial membrane so that the cycle can re-start. Although many players involved in all the steps required for vesicular trafficking have been identified, including molecular motors, tethers and soluble NSF (*N*-ethylmaleimide sensitive fusion proteins) attachment receptors (SNAREs), the complete picture on Rab-mediated transport across membranes is far from complete, with many unresolved questions (Box 1).

Box 1 **Outstanding questions in Rab GTPase regulation during membrane trafficking.**

Challenges in the field include:

1. Defining the chronology of Rab cascades;
2. Quantifying the number and diversity of Rab-mediated molecular interactions when bound to GDP or GTP;
3. Identifying regulatory mechanisms, including GEFs and GAPs;
4. Exploring the crosstalk between signaling/metabolic pathways and Rab function;
5. Determining the kinetics and composition of sequential protein scaffolds coordinating movement, tethering and fusion events.

One intriguing issue relates to the mechanisms assuring transport directionality across several sub-organelles. In recent years, it was identified that protein networks are reversibly and sequentially assembled employing “Rab conversion cascades” (Figure 2B), where effectors for one Rab execute a GEF or GAP (or both) function downstream (examples in Box 2) [58,72,73,74,75,76,77,78,79]. This mechanism might also be employed in positive (negative) feedback loops when the effector is the GEFs (GAP) for the initial Rab. This establishes a chronological order for sequential recruitment (and inactivation) of Rabs, and thus maximizes the directionality of trafficking.

**Figure 4:**
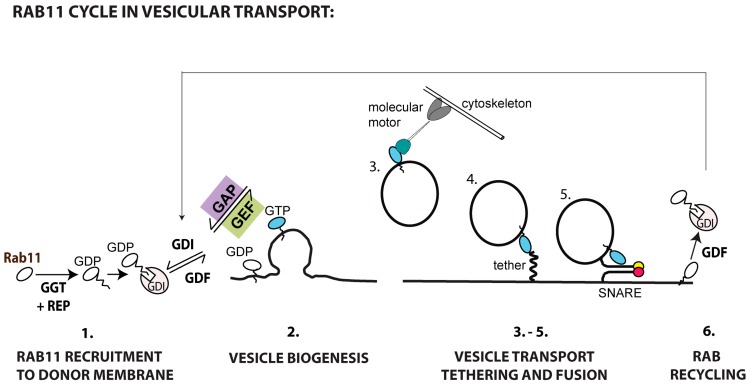
Rab11 cycle in vesicular transport (steps in Rab11 cycle are numbered from 1 to 6).

**Figure 5:**
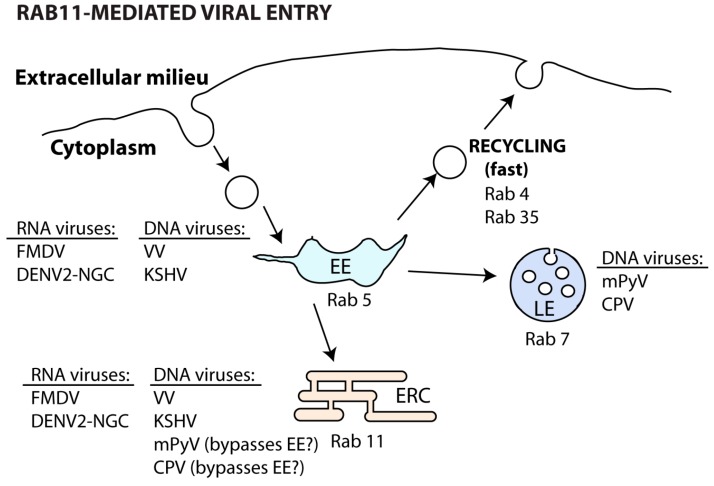
Involvement of the ERC in viral entry in the host cell. Upon entry, viruses can be trafficked from the EE to the ERC or to LE. Others can bypass EE and be directed to the ERC for viral uncoating.

Rab activation can, in addition, be regulated by post-translational modifications that interfere with Rab function (Figure 2A-4), with some examples identified in yeast and vertebrates, but the most notorious occurring in bacterial infection [80,81,82,83,84,85]. Whether these Rab modifications take place during viral infection has not been reported. This type of regulation could, in principle, allow the integration of signaling pathways and metabolism in vesicular trafficking in response to physiological cues, via activation of kinases and phosphatases, for example, but so far experimental evidence is scarce [86]. A metabolic related factor shown to interfere with Rab deactivation was the cellular level of cholesterol. When in high levels, cholesterol was proposed to reduce Rab extraction from membranes through the inhibition of the activity of the corresponding GDI (Figure 2A-5) [87,88,89], a topic explored in Section 4.3.

As a final remark, Rabs have been found in a complex in the GDP form, which suggests that depending on the GDP/GTP status Rabs could be part of distinct functional complexes. Although requiring further support, this hypothesis suggests that Rabs might also function when in an off status [90]. In agreement, GDP-Rab11 was found associated with protrudin, a protein able to bind to the plus-end motor kinesin II [91].

Box 2 **Rab cascades or feed-forward loops involving GEFs and GAPs.**

Examples of Rab-mediated recruitment of effectors with GEF or GAF activity for other Rabs.

**Table:** 

Cascades Involving	Rab A	GEF/GAP B	Rab B	References
GEFs	Rab11-GTP	Rabin8	Rab8-GTP	[58,72,73,74]
	Rab33-GTP	Ric-Rgp	Rab6-GTP	[79]
	Rab32/38-GTP	VARP	Rab21-GTP	[76,77]
GAPs	Rab9	RUTBC1	Rab32	[78]
	Rab9	RUTBC2	Rab36	[79]

In conclusion, there is a vast array of mechanisms regulating membrane identity that could be targeted or altered during viral infection, but thus far have not been investigated.

### 3.2. Phosphoinositide Code

Many of the dynamic processes of recruitment, activation or extraction of proteins from membranes have also been associated with specific phosphoinositides, which provide reversible docking sites for regulatory factors. They are a family of evolutionary conserved phosphorylated derivatives of phosphatidylinositol (PI). As represented in Figure 1B, these lipids result from the reversible phosphorylation of hydroxyl groups bound to carbon 3, 4 and 5, giving rise to seven forms that bind to the cytosolic side of membranes via two fatty acids esterified to carbons 1 and 2 of the glycerol moiety [92].

Seminal studies have shown that proteins contain binding domains for specific phosphoinositides located in distinct membrane compartments (Figure 1A and [93,94,95,96]). How phosphoinositides acquire defined localizations is not completely elucidated, but depends on the enzymatic activity of transiently recruited kinases and phosphatases able to convert one isoform into another (Figure 1B and reviewed in [38,92,97]). Minor species of phosphoinositides can, however, be found in each membrane, forming functional microdomains [98,99,100]. For example, during the formation of macropinosomes, highly dynamic actin-containing ruffles transform into circular cups, closing subsequently at their terminal edges to create endosomes [101]. These alterations correspond to acquisition and loss of several complexes transiently associated with the membrane that have been spatially and temporally resolved. Confined to circular ruffles, that are considered functional microdomains, sequential peaks of phosphatidylinositol-4,5-biphosphate (PI4,5P2), phosphatidylinositol-3,4,5- triphosphate (PI3,4,5P3), phosphatidylinositol-3-phosphate (PI3P), diacylglycerol, phosphatidylinositol-3,4-biphosphate (PI3,4P2), precede the activities of protein kinase C-alpha, Rac1, Ras and Rab5 [102]. These findings strongly suggest that the regulation of phosphoinositides and proteins is intertwined, with some upstream factors regulating the location and function of subsequent events, and this will be explored next.

#### 3.2.1. Phosphoinositide Feed-Forward Loops and Cascades

Phosphoinositide cascades including phosphoinositide-mediated recruitment of kinases and phosphatases have been reported, and shown to regulate downstream membrane trafficking steps (Figure 3). It is the case of the phosphatidylinositol 3-phosphate 5-kinase PIKFYVE (Phosphoinositide kinase, FYVE (Fab 1, YOTB, Vac 1 and EEA1) finger containing) that binds to PI3P domains on early endosomes [103,104]. Downstream (in late endosomes), this enzyme will catalyze the synthesis of phosphatidylinositol-3,5-biphosphate (PI3,5P2) [105,106]. Interestingly, in yeast, the known regulators of PI3,5P2 levels, Fab1p and phosphatase Fig4p, directly contact with Vac14p, which functions as a complex scaffold [107].

#### 3.2.2. Phosphoinositide and Rab Crosstalk

A series of Rabs and their effectors was found to regulate phosphoinositide metabolism and vice-versa. The first example comes from the capacity of Rab5 to bind the β-isoform of PI3P kinase, PI3Kβ, as well as phosphatidylinositol-4-phosphate (PI4P) and phosphatidylinositol-5-phosphate (PI5P) phosphatases [26]. In addition, both Rabs and phosphoinositides cooperate by coincidence detection, a process in which lipids and proteins together and not isolated perform a function (as reviewed in [50]) to co-coordinate trafficking events. The well-known role of Rab5 and PI3P in recruiting early endosome antigen 1 (EEA1), a factor involved in membrane tethering, is an example of this [108,109]. Finally, complex regulatory networks between phosphoinositides, Rab or ARF GEFs and GAPs, or their cognate effectors, were shown to provide sequential docking platforms, maximizing efficiency in trafficking routes. In pancreatic beta cells, PI3P kinase-localised production of PI3,4,5P3 provides an anchor site for the ARF GEF ARNO. ARNO in turn displays GEF activity for ARF6 and remodels actin during endocytosis [110,111]. Furthermore, in the presence of glucose, ARNO binds the GAP for Rab27a, the latter being involved in exocytosis. This intricate crosstalk between phosphatidylinositol phosphate (PIP), ARFs and Rabs responds at least partially to cellular demands of endocytosis and exocytosis [112].

In conclusion, both phosphoinositides and Rab GTPases cooperate to coordinate trafficking events, via sequential dynamic reactions and cascades, contributing to spatiotemporal regulation, maturation and function of endocytic compartments [38,92,101,113,114]. The crosstalk between these groups is starting to emerge in the cell biology field, but remains completely unexplored in viral infection. The way viruses interfere with these communication systems will help to decipher the order by which these cascades operate.

## 4. The Rab11 Centric View of the ERC

The examples provided elucidate the vast number of networks and the complexity of the circuitry involved in trafficking. We will now discuss the biology underlying cargo transport to the plasma membrane via the ERC, to highlight the number of molecules available for viral targeting. The ERC is defined by the presence of Rab11 and its effectors [36,37]. Rab11 regulates recycling from the ERC and transport of cargo from the TGN to the plasma membrane [22,23,24]. It is important to note, however, that there are other routes for TGN transport to the surface [22,23,24]. Rab11 comprises the products of three genes Rab11a, Rab11b and Rab25 [12,90]. Rab11a and Rab11b share 89% amino acid identity, whereas Rab11a or Rab11b share 61% or 66% identity with Rab25, respectively. The three isoforms have been implicated in human disease and infection. Rab25 has been found in high levels in a number of cancers (reviewed in [115]) and was shown to contribute to invasiveness of cancer cells by promoting integrin trafficking [116]. The present review explores the roles of the ERC in viral infection, which have been linked to Rab11a and to a lesser extent to Rab11b and Rab25. For this reason, Rab11a will be almost exclusively explored henceforward.

Figure 4 shows the sequential steps involved in Rab11a-mediated transport to the plasma membrane. Rab11 is recruited to and activated in membranes (step 1). Whether all Rab11 recruited is activated during vesicular biogenesis, or alternatively a pool is reserved for posterior activation close to the acceptor membrane to mediate tethering and fusion, remains to be elucidated (step 2). When active, Rab11 attracts effectors responsible for transporting cargo along cytoskeletal tracks (step 3), tethering (step 4) and fusing vesicles (step 5) with the plasma membrane. For termination, Rab11 is switched off by GAPs and recycled to the original membrane, associated with GDIs, and the process can re-start (step 6). The complete set of molecular interactions required for each of these steps is unavailable for most cargo requiring Rab11-mediated transport (Table 1 and outstanding questions highlighted in Box 1). A notable exception is ciliogenesis, where transitory complexes and regulatory cascades have been elucidated [74,117]. The state-of-the-art on players and regulation involved in Rab11-mediated transport follows (Table 1).

### 4.1. Rab11 Membrane Recruitment and Activation

Mechanisms underlying Rab11 recruitment to membranes are far from elucidated. Rab11 is prenylated at the C-termini for insertion in membranes [118], and two Rab11-GDIs (alpha and beta) were identified as Rab11 binding partners [119]. The lipid modifying enzyme phosphatidylinositol 4-phosphate kinase type III (PIP4KIII), which is located in the Golgi and catalyzes the synthesis of PI4P, was shown to recruit Rab11 and its effectors, however its role in the ERC homeostasis has not been explored [120,121]. Interestingly, besides minor amounts of PI4,5P2 and PI3,4,5P3 [122,123] (highlighted in red in Figure 1B), the ERC has not been reported to contain PI4P, and therefore additional unidentified PIP modifications will be required for membrane maturation. Two *bona fine* Rab11-GEFs have been identified so far. Crag, a differentially expressed in normal and neoplastic cells (DENN) protein, was the first Rab11-GEF identified. Crag is required for trafficking of rhodopsin from the TGN to the plasma membrane in *Drosophila* photoreceptor cells via a Rab11-dependent vesicular transport [124]. DENND4A, B and C are the three human homologues of Crag and their role in promoting Rab11 GDP-to-GTP exchange in humans awaits clarification. Recently, the *C. elegans* protein REI-1 and its human homologue SH3-domain binding protein 5 (SH3BP5) were demonstrated to act as Rab11-GEFs *in vitro* [125]. In hippocampal neurons, huntingtin and lemur kinase 1/ apoptosis-associated tyrosine kinase 1 (LMTK1/AATYK1) containing complexes were shown to positively regulate the GDP/GTP exchange rate of Rab11 in pull down assays. However, as Rab11 and huntingtin do not interact directly, it was hypothesized that these proteins co-precipitated in a complex along with an unidentified Rab11-GEF, but this requires experimental support [126,127]. As mentioned above, recruitment of Rab11 was shown to bypass Rab GEFs during rhodopsin transport in *Drosophila* eye [59]. Posttranslational modifications were shown to modify Rab activity, but no posttranslational modification was reported for Rab11 so far.

In sum, identified factors involved in Rab11 recruitment and activation are not numerous and their usage is far from clear. It is possible that more will be identified. Still, a component that is missing is how these factors are themselves regulated in response to cellular stimuli as metabolic, infectious and stress conditions. This might help to understand trafficking at the ERC, human disease and infection.

### 4.2. Rab11 Recruitment of Effectors

A comprehensive list of identified Rab11 effectors can be found elsewhere [12,90]. Here, we will focus on effectors promoting crosstalk with other GTPases and allowing interaction of ERC with other arms of the endomembrane system, the cytoskeleton or involved in tethering and fusion to the plasma membrane (Table 1).

#### 4.2.1. Rab11 Effectors Involved in Rab Cascades

The Rab11A-Rabin8-Rab8 cascade, illustrated in Box 2, is not an isolated mechanism for Rab11 crosstalk and coupling to other membranes. Rab3a interacting protein (rabin3)-like 1, a GEF for Rab3a, Rab8a and Rab8b, was identified as an effector for Rab11a and b, coupling Rab3 or Rab8 with Rab11a trafficking [147]. Another example refers to recycling of proteins reaching the ERC from TGN via a retrograde transport, which has been associated with Rab6. Recently, one of its effectors, Rab6-interacting protein 1, was shown to interact with Rab11a in its GTP-bound conformation, coupling these two systems [148].

#### 4.2.2. The Crosstalk between Rab11 and the Cytoskeleton

Rab11 vesicles are transported on cytoskeletal tracks within the cell, moving towards or away from the microtubule-organizing center (MTOC), associating with a series of different molecular motors. Effectors can recruit distinct molecular motors to transport cargo either on microtubules using dyneins (minus-ends) and kinesins (plus-ends) or on actin using myosins. Some molecular motors were shown to require adaptor proteins for Rab11 binding. The adaptors include the Rab11-family interacting proteins (FIPs), with several members identified, numbered 1–5, and additionally diversified by three isoforms of FIP1, differentiated as A–C [149,150,151,152,153,154]. The best studied triad consists of Rab11-FIP2-MyoVb [128,129,130,131,132]. MyoVb recruitment serves a dual purpose: it tethers recycling endosomes to actin at the microtubule–actin junction [130] and also coordinates delivery to cell surface [155]. Other pairs of effector-molecular motors identified are: Rab11-FIP5-Kinesin II for plus-end movement [136], and Rab11-FIP3-DLIC-1 and 2 for minus-end directed transport [146]. As all identified FIPs share a Rab11 binding domain [150], they must compete for Rab11 access. Therefore, a point that deserves attention is how each effector is selected for Rab11 binding. Phosphorylation of at least FIP2 and FIP3 could regulate their engagement in recycling [156,157], but much needs to be explored in this context. Yeast two hybrid and fluorescent resonance energy transfer studies suggest that some molecular motors might bind Rab11 without the aid of adaptors. This is the case of Kinesin KIF13A [143], a molecular motor implicated in the recycling of avb3 integrins [158] and mannose-6-phosphate receptor [159].

#### 4.2.3. Rab11 Vesicular Targeting and Fusion with the Target Membrane

As Rab11 vesicles reach the target membrane, they need to tether and fuse with it. This process involves the binding of proteins in vesicles to their cognate partners located in acceptor membranes (reviewed in [160]). Sec15 is a tether involved in Rab11a vesicular docking to the target membrane and belongs to the exocyst, an eight polypeptide complex involved in constitutive secretion [137,138,139,140]. Interestingly, Rab11b has been proposed to work as a membrane tether, although this is still controversial [133]. Recently, the docking factor Munc13-4, proposed to interact with soluble NSF attachment protein receptors (SNAREs) [144], was shown to bind Rab11 and promote tethering between vesicles and membranes [161]. Finally, several members of the SNARE family have been associated with Rab11-mediated trafficking: SNAP25, the vesicle-SNARE component that binds to the exocyst [134]; SYN4 target-SNARE, which is required for cortical granule exocytosis after fertilization of *C. elegans* oocytes [141]; and recently, VAMP8, a vesicle-associated membrane protein, that in conjunction with the vesicle-SNARE syntaxin11 promotes cytotoxic granule fusion at immune synapses in primary human T lymphocytes [145].

### 4.3. Rab11 Inactivation and Recycling

Solely proteins containing a TBC (Tre-2, BUB2p, Cdc16p) domain have thus far been identified as Rab11a GAPs. One has been identified in flies, Evi5, and regulates border cell migration during *Drosophila* oocyte development [135]. However, its Rab11a-GAP homologue in mammalian cells is not clear, with controversial reports coming to opposite conclusions [162,163]. Another TBC-containing protein, TBC1D9B, has recently been identified in polarized MDCK cells as catalyzing GTP hydrolysis of Rab11a (Figure 2A-2), and its depletion negatively impacted the transcytosis of immunoglobulin A [142].

Recycling of Rab11a must occur via GDIs. Interestingly, studies have also reported that the levels of cellular cholesterol and Rab11 distribution were intertwined, with an increase in the levels of activated Rab11 resulting in cholesterol accumulation in ERC [87,88]. Such co-localization was proposed to correlate to inhibition of Rab11 extraction from membranes by GDI (Figure 2A-5), which was also observed for Rab9 in late endosomes [89].

A lot of progress has been made in identifying players involved in Rab11-mediated trafficking, especially in the binding of Rab11 vesicles to molecular motors, allowing the investigation of more challenging questions. Apart from the highlighted topics in Box 1, other outstanding issues relevant to Rab11 include the understanding of how and in what situation each effector is recruited; the determination of the composition of each transiently assembled complex in ERC microdomains; and a detailed elucidation of crosstalk, feed-forward and feedback loops operating in this system.

Viral usage of the ERC will, in principle, impact function, as during infection an additional number of molecular interactions must be established in an already densely populated environment. In fact, alterations to ERC distribution have been reported during infection [164,165], as well as ERC-mediated roles in host cytoskeleton structure [166,167] and immune responses [168], but whether there is a causal relationship between the two parameters is unclear.

## 5. Involvement of ERC in Viral Lifecycles

A plethora of evidence implicates the recycling endosome and its major regulator, Rab11, in several stages of the lifecycle of distinct viruses (Table 2 and Table 3 and discussed in detail below).

### 5.1. Viral Entrance

Both DNA [169,170,171] and RNA [172,173] viruses can use the slow recycling endosome during internalization steps (Table 2 and Figure 5).

Binding of vaccinia virus (VV) to the host cell receptors integrin β1 and CD98, both normally recycled in a Rab11 and Rab22-regulated manner, allows virus entry in macropinosomes and trafficking to EE (Rab5). Here, endosome fission and sorting of virus-containing vesicles to recycling endosomes (Rab11 and Rab22) allows viral fusion and core uncoating [169]. Likewise, the Kaposi’s sarcoma-associated herpesvirus (KSHV) particles also co-localize with early and recycling endosomes soon upon entry [174]. Interestingly, other DNA viruses, such as mouse polyomavirus (mPyV) [170] and canine parvovirus (CPV) [171], bypass the EE soon after endocytosis. In both cases, recently internalized viruses can be found in the perinuclear ERC [170,171], but they are later trafficked to late endosomes, lysosomes and ER to promote viral uncoating [171,175]. However, the passage of incoming mPyV through the ERC seems to be non-productive and may be related to recycling of viral material back to the surface [175].

Similarly, RNA viruses can be internalized and initially delivered to Rab5-controlled compartments. In the case of foot-and-mouth disease virus (FMDV), a small proportion of viral particles also end up in Rab11 compartments [173]. Given the virus sensitivity to acidic environments, it is possible that FMDV increases the chance of establishing infection by avoiding the degradative pathway.

Regardless of the entry route, dengue viruses (DENV) are first transported to EE (Rab5) and then sorted differently depending on the strain. Some strains (DENV2-16681) follow the degradative pathway (Rab7), whereas others (DENV2-NGC) are directed to the recycling pathway (Rab22 and Rab11) for membrane fusion and nucleocapsid release into the cytoplasm. The determinants of this discrepant vesicular sorting between DENV strains are not currently established. However, it has been proposed that trafficking to distinct endosomes may be related to their specific membrane lipid composition, facilitating viral fusion of the different strains [172].

Overall, viral usage of the ERC during entry in the host cell may be related to: (1) endocytosis through binding to receptors that are recycled by Rab11 and Rab22; (2) lipid membrane content that promotes host and viral membrane fusion for uncoating and (3) escape from degradation in late endosomes and lysosomes.

### 5.2. Viral Assembly

The ERC is pivotal for the cytosolic transport of *de novo* synthesized RNA particles of viruses from the *Paramyxoviridae* [164,176,177,178], *Orthomyxoviridae* [165,179,180,181,182] and *Bunyaviridae* [183] families (Table 3). Independently of the replication site, viral ribonucleoproteins (vRNPs) traffic attached to vesicles, positive for Rab11, towards assembly and/or budding zones, facing the cytosol. It is still not fully understood how vRNPs attach to these vesicles. In the case of IAV, Rab11 binding requires the viral polymerase subunit PB2, but whether this binding is direct or mediated by another host/viral factor, remains to be determined [165,179,181]. In all cases, the association between vRNPs and Rab11 seems to occur only with the Rab11 active form. As infection progresses, vRNPs from Sendai virus (SeV) [164,176], human parainfluenza virus type 1 (hPIV1) [164] and IAV [165,179,181,182] accumulate in large cytoplasmic Rab11-aggregates. Their function, if any, has not been elucidated, but the observation that several unrelated viruses are able to induce Rab11 redistribution, strongly indicates that aggregates play a role in infection. Aggregates could function as platforms for concentrating viral components for viral assembly, but this hypothesis requires scientific support. In line with this, IAV has a segmented genome composed of eight independent vRNPs that need to be packaged to form infectious particles. Most IAV virions contain no more than eight segments, which strongly supports that there is a selective process mediating assembly. Interestingly, it has been reported that the lack of a functional Rab11 leads to a decrease in the co-localization of segments in the cytoplasm [184], which could affect vRNP core formation [184,185].

Apart from genome transport, other roles have been assigned to the ERC, which include capsid envelopment [186] and delivery of viral proteins to assembly sites [187,188,189]. In fact, the ERC has been recently implicated in the envelopment of herpes simplex virus-1 (HSV-1) capsids. The envelope is composed of membranes containing viral glycoproteins that are internalized from the plasma membrane via Rab5- and Rab11-regulated pathways [186]. In accordance, the fusion protein of Nipah virus (NiV), expressed as a precursor at the plasma membrane, is proteolytically activated by cathepsin B in the early (Rab4) and late (Rab11) recycling compartments. It is subsequently recycled to the surface for incorporation in the budding virions, a key step to ensure successful cell-to-cell spread by NiV [187]. Similarly, the inclusion of the envelope glycoprotein complex (Env) onto the developing particle is a crucial step in the lifecycle of human immunodeficiency virus 1 (HIV-1). For this virus, Env trafficking from the ERC to the surface assembly sites is mediated by FIP1C bound to Rab14 and not Rab11 [188]. The HIV-1 accessory protein Vpu also seems to require recycling through Rab11-vesicles in order to counteract host factors that restrict particle assembly/release [189].

### 5.3. Viral Budding and Release

A potential role for the ERC in viral egress has been described for both positive- and negative-sense RNA viruses of the *Paramyxoviridae* [190,191], *Orthomyxoviridae* [180], *Retroviridae* [192,193,194] and *Flaviviridae* [195] families (Table 3).

For the *Paramyxoviridae* [190,191] and *Orthomyxoviridae* [180] viruses, the ERC influences budding at the surface. For these viruses, virion egress involves induction of membrane curvature, evagination, formation of a neck and scission to release particles. The lack of Rab11 has been associated with morphologically aberrant budding virions that present large necks and fail to scisse. The Rab11 effectors FIP1 and FIP2 have been shown to facilitate fission of respiratory syncytial virus (RSV) from the apical side of the plasma membrane [190,191]. Moreover, FIP2 regulates the filament length of the RSV budding virion [191]. Similarly, the Rab11 pathway is also hijacked for efficient budding of both spherical and filamentous IAV virions [180]. Additionally, FIP3 specifically regulates filamentous, but not spherical, virion release from the surface [180]. Given that Rab11, as explained above, regulates several steps in vesicle trafficking, it is unclear for most of these viruses which are the factors downstream of Rab11 and/or of FIPs that promote budding.

**Figure 6:**
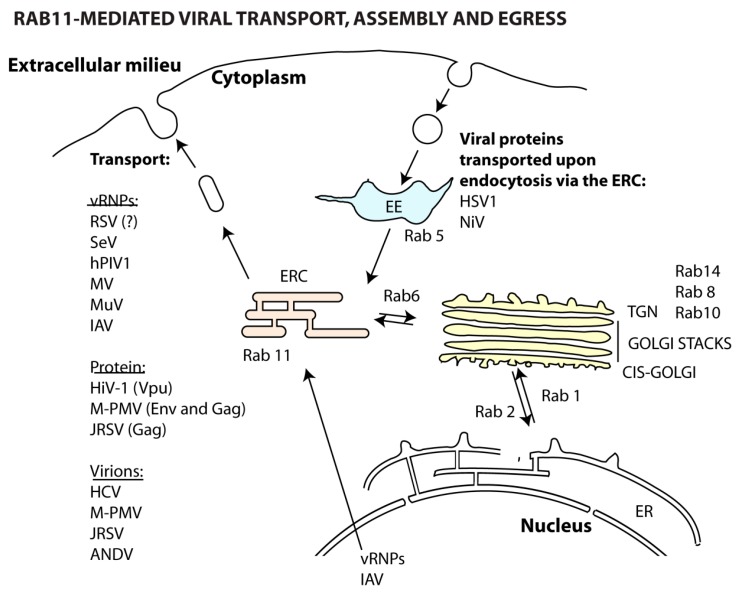
Involvement of the ERC in viral assembly, budding and release. The ERC can be used by different viruses for transport of viral genome particles and proteins towards assembly sites. Moreover, it is also involved in the transport of entire virions to the cell surface.

The recycling pathway is also involved in the transport of assembled capsids or virions towards the plasma membrane of viruses belonging to the *Retroviridae* [192,193,194,196] and *Flaviviridae* [195] families (Figure 6). The Gag-containing capsids of two retroviruses, Mason-Pfizer monkey virus (M-PMV) [192,196] and Jaagsiekte sheep retrovirus (JRSV) [193], are assembled at the MTOC and exit efficiently the cell via the ERC. In the case of M-PMV, the immature capsids are co-trafficked with Env-recycling endosome vesicles en route to the plasma membrane [192,196]. Also, the assembled capsids of hepatitis C virus (HCV) are enveloped and matured in the ER and Golgi/TGN compartments, respectively, and complete particles then reach the surface on Rab11-endosomes [195]. The molecular players involved in this transport, including molecular motors, have not been identified. Finally, the ERC facilitates transcytosis of intact HIV-1 virions that enter vaginal epithelial cells through endocytosis [194].

## 6. Cellular Alterations Resulting from the Interplay between the ERC and Viruses

### 6.1. Cytoskeleton Alterations

Reorganization of the host cell cytoskeleton is a common feature during viral infection. Both actin and microtubule tracks can be used for the circulation of viruses and viral components through the ERC.

Actin dynamics has been implicated in the cell entry and delivery of KSHV and VV particles to the ERC [169,174]. However, the machinery involved in these steps is not yet well defined. It is known that the WASH-VPEF-retromer complex, which controls actin polymerization at endosomes, supports the sorting of incoming VV from EE to ERC [169]. Vesicular transport of input virus in this direction can also occur along microtubule tracks, mediated by the dynein motor, as is the case for CPV [170] and mPyV [175]. Interestingly, the majority of FMDV is uncoated at the EE and only a modest sub-population follows to the ERC to complete this process. Hence, it is not surprising that inhibition of microtubule-dependent transport does not significantly impair FMDV infection [173].

Several reports consistently implicate usage of the cytoskeleton during late stages of viral infection. The newly replicated genome of SeV [176], IAV [165,179,181], MV [178] and MuV [177] is carried by Rab11-vesicles along microtubules towards assembly sites, albeit the Rab11 adaptors and molecular motors required for this process remain unidentified. Whether this transport is somehow different from that observed in uninfected cells is not clear. However, it is known that Rab11 distribution changes with infection with some of these viruses (SeV and IAV), suggesting alteration in sorting efficiency that could relate to vesicular movement, or alternatively in downstream tethering and fusion events. To our knowledge, Y-box-binding protein 1 is the only cellular protein found to recruit IAV progeny vRNPs to microtubules, thereby facilitating their travel onto Rab11-vesicles [197]. Recent data suggests that SeV vRNPs can also be transported by the molecular motors myosin Vb and Vc on actin filaments [164]. The vRNP-Rab11 trafficking is a complex process expected to require both types of cytoskeleton, with microtubules and actin tracks being used for long and short-range movement, respectively. In fact, Rab11 is recognizably able to interact with different adaptors and motors to enable vesicle movement [90]. In line with this, the Rab11 effectors—FIP1/FIP2 adaptors and myosin Vb motor—are required for the assembly and budding of RSV at the apical surface [190,191]. Nevertheless, a direct role for this ternary complex in RSV genome trafficking has not been demonstrated experimentally. Furthermore, distinct retroviral proteins that traffic by the Rab11-recycling compartment require either actin (myosin Vb)-based transport, like HIV-1 Vpu [189], or microtubule-dependent transport, such as M-PMV Env and Gag [192]. Interestingly, JRSV Gag protein reaches the pericentriolar ERC via dynein movement along microtubules [193]. Also, viral proteins can manipulate the concerted action between the host cytoskeleton and vesicular trafficking to optimize viral exit and spread, as is the case of the human adenovirus E4orf4 protein. This protein hijacks the host Src family kinases-signaling pathway to trigger a dramatic mobilization of the ERC [166,167]. On the one hand, E4orf4 fosters the actin-regulated transport of Rab11 vesicles to the Golgi [166]. On the other hand, it can induce a Rab11-dependent mobilization of mitochondria to the vicinity of a polarized actin network [167]. Hence, Rab11 likely coordinates the polarized vesicular trafficking, cytoskeleton dynamics and organelle functions during viral infection.

Although not totally clear, Rab11, PI4,5P2 and several actin regulators (Exo70, N-WASP and ezrin) present in F-actin-rich nanotubes may promote the dissemination of HIV-1 between contacting T cells [198]. As mentioned before, entirely completed viral particles can egress the infected cell. Enveloped HCV particles do so by trafficking on Rab11 vesicles along the microtubule network [195].

### 6.2. Host Antiviral Response

Information regarding a role for the ERC in the immune response to viruses is scarce. Rab11 has been implicated in the deployment of the T cell co-stimulatory molecules CD80 and CD86 from the cell surface of monocytes, during HIV-1 infection. The HIV-1 Nef protein re-routes these proteins from the cell surface to the Golgi in vesicles containing Rab11 as a viral immune evasion strategy [168].

### 6.3. Membrane Remodeling

Interesting emerging reports disclose alterations of the ERC membranes during viral infection. Thus far, two types of changes have been reported. One, during IAV infection, in which cholesterol was shown to accumulate at the ERC to promote raft formation at the plasma membrane, from where virions bud [61]. The second, detected for infections with several viruses as SeV [164,176], hPIV1 [164] and IAV [165,179,181,182], in which Rab11 dramatically redistributes from discrete puncta to large aggregates, of unknown nature and formed by an uncharacterized process. Being a common feature of several viruses, the changes in Rab11 distribution might be functional and, hence, important to characterize. In the case of IAV, the levels of activated Rab11, the form bound to membranes, were shown to increase during infection. Increase in Rab11 activation has previously been related to the appearance of large structures in the cytoplasm [199]. Interestingly, the increase observed during IAV infection was hypothesized to be due to viral induction of an yet unidentified GEF [61]. No other mechanism has been reported for any of the other viruses, and thus, much needs to be explored to understand the interplay between the ERC and viral infections.

## 7. Future Perspectives

Much has been done over the last decade to clarify mechanisms involved in vesicular trafficking, namely in the establishment of membrane identity and regulatory processes maximizing sequential progression of cargo. With the identification of specific factors defining sub-compartments and the availability of genetic tools to target protein expression, many of these factors were found to be crucial for a plethora of viral infections. In this context, Rab11 emerged as a prominent host requirement for an increasing number of steps and viruses. Given that Rab11 is part of different complexes and pathways, a challenging aspect for the future is defining which of these scaffolds and their regulatory mechanisms are required for the different viruses, and what happens to the remainder of the processes under Rab11 control, especially from the host side. In this sense, it is pertinent to identify GEFs and GAPs, molecular motors, tethers and SNARES regulating Rab11 function of each viral infection. In addition, there is not much information relating phosphoinositides, Rab11 and viral infection, which deserves some attention. Furthermore, viral targeting of the ERC might alter its architecture, function and impact in the physiological processes it controls in the cell. A challenge is to characterize at an ultrastructural level the changes of the ERC during infection with IAV, SeV and hP1V1 (and possible others), to obtain insights into the regulatory cascades operating during Rab11 trafficking. Finally, defining Rab11 complexes involved in viral entrance or exit, offers a unique opportunity to differentiate constitutive recycling *vs.* inducible secretory pathways under Rab11 control that occur in the uninfected cell.

## Figures and Tables

**Table 1:** Modulators and effectors identified in Rab11 cycle in vesicular transport.

Recruitment/Activation	Transport	Tethering	Fusion	Inactivation/Recycling
Crag [124]	Myosin Vb (FIP2) [128,129,130,131,132]	Rab11b [133]	SNAP25 [134]	Evi5 [135]
REI-1 [125]	KIF5a and KIF3 (Kinesin II) (FIP5) [136]	Sec15 (exocyst) [137,138,139,140]	SYN4 [141]	TBC1D9B [142]
PIP4KIII [120,121]	KIF13A [143]	Munc 13-4 [144]	VAMP8 [145]	Cholesterol [87,88,89]
	DLIC1/2 * (FIP3) [146]			

* Binding of this effector to Rab11 has been shown to be indirect via Rab11-family interacting proteins (FIPs). Crag: CRMP5-associated GTPase; REI: RAB-11-interacting protein; PIP4KIII: phosphatidylinositol 4-phosphate kinase type III; KIF: Kinesin family member; DLIC: Dynein light chain; Rab: Ras-related in brain; Munc: mammalian uncoordinated; SNAP: Synaptosomal-associated protein; SYN: syntaxin; VAMP: vesicle-associated membrane protein; Evi: Ecotropic Viral Integration Site; TBC: Tre-2, BUB2p, Cdc16p.

**Table 2:** Evidence for viral usage of the ERC at entry steps.

Family	Genome	Virus	Host	Cell Type	Pathology	Evidence for Usage of ERC	References
*Poxviridae*	ds DNA	Vaccinia virus (VV)	human and a wide range of animals	Keratinocytes, dermal fibroblasts and microvascular endothelial cells; tropism for tumour cells	Similar to smallpox, but milder; causes rash, fever, headache and body aches	Virus uncoating into the cytoplasm occurs in Rab11- and Rab22-positive recycling endosomes	[169]
*Polyomaviridae*	ds DNA	Mouse polyomavirus (mPyV)	mouse	Epithelial and mesenchymal cells of the respiratory system, kidneys and brain	Induce carcinogenesis	Partial co-localization of capsids with the Rab11-recycling endosomes upon entry	[170]
*Herpesviridae*	ds DNA	Kaposi’s sarcoma-associated herpesvirus (KSHV)	human	B lymphocytes, endothelial, and epithelial cells	Kaposi’s sarcoma, peripheral effusion lymphoma or multicentric Castleman’s disease	Co-localization of viral particles with Rab11-endosomes early after entry	[174]
*Parvovirus*	ss DNA	Canine parvovirus (CPV)	dog	Rapidly dividing lymphocytes and epithelium of the small intestine	Lethargy, vomiting, fever, and diarrhea	Co-localization of capsids with transferrin in a perinuclear area soon after internalization	[171]
*Flaviviridae*	+ ss RNA	Dengue virus (DENV)	human	Keratinocytes, Langerhans cells, monocytes and macrophages	Mild fever; complications like dengue hemorrhagic fever may occur (fever, damage to lymphatic and circulatory system)	The recycling pathway (Rab22 and Rab11) is used for viral fusion and uncoating steps	[172]
*Picornaviridae*	+ ss RNA	Foot-and-mouth disease virus (FMDV)	cattle, pig, sheep and goat	Epithelia	Fever, followed by blisters in the mouth and feet that may rupture and lead to lameness	Capacity to infect cells dependent on active Rab11	[173]

ds: double-stranded; ss: single-stranded.

**Table 3:** Evidence for viral usage of the ERC at assembly and budding steps.

Family	Genome	Virus	Host	Cell Type	Pathology	Evidence for Usage of ERC	References
*Paramyxoviridae*	− ss RNA	Respiratory syncytial virus (RSV)	human	lung epithelia	upper and lower respiratory tract infections (such as colds, bronchiolitis and pneumonia)	Rab11-FIP1 and -FIP2 are required for virus replication and budding at the apical plasma membrane	[190,191]
		Sendai virus (SeV)	rodent	lung epithelia	respiratory tract infection	Rab11a and transferrin co-localize with vRNPs in large intracellular aggregates	[164,176]
		Human parainfluenza virus type 1 (hPIV1)	human	lung epithelia	respiratory tract infection; croup or pneumonia	Rab11a co-localizes with vRNPs in large intracellular aggregates	[164]
		Measles virus (MV)	human	lung tissue macrophages and dendritic cells, lymph node B and T cells, epithelial cells of the liver, spleen or even brain	immune system infection and respiratory tract infection	RNPs co-traffic with Rab11a endosomes, accumulate at the apical recycling compartment and beneath the apical membrane	[178]
		Mumps virus (MuV)	human	systemic epithelia	swelling of parotid glands, accompanied by severe complications such as orchitis, aseptic meningitis, pancreatitis and deafness	Rab11 recycling endosomes transport vRNPs to the apical membrane	[177]
		Nipah virus (NiV)	human and animals	systemic epithelia and endothelia	severe encephalitic and respiratory diseases	Early (Rab4) and late (Rab11) recycling endosomes are likely involved in the cleavage and activation of F protein	[187]
*Orthomyxoviridae*	− ss RNA (8 segments)	Influenza A virus (IAV)	human, pig, horse, bird	lung epithelia	respiratory tract infection	Rab11 is required for vRNP trafficking and virion budding at the surface; interaction between vRNPs and Rab11 is mediated by viral PB2; Rab11-FIPs influence vRNPs localization at the recycling endosome and later at the surface; Rab11-FIP3 required for filamentous virion formation	[165,179,180,181,182]
*Bunyaviridae*	− ss RNA (3 segments)	Andes virus (ANDV)	humans and rodents	endothelia	hantavirus cardiopulmonary syndrome (HPS)	Rab11 is required for viral production and co-localizes with nucleocapsid protein	[183]
*Retroviridae*	+ ss RNA (2 copies)	Mason-Pfizer monkey virus (M-PMV)	macaque	epithelial	fatal immunodeficiency syndrome	Recycling endosome (Rab11) required for the Env-dependent export of Gag-assembled capsids towards the surface	[192]
		Jaagsiekte sheep retrovirus (JRSV)	sheep and goat	lung epithelia, lymphocytes and myeloid cells	ovine pulmonary adenocarcinoma (OPA)	Recycling endosomes (Rab11) co-localize with Gag protein in the pericentriolar region and are involved in virion cell exit	[193]
		Human immunodeficiency virus 1 (HIV-1)	human	CD4+ T cells, macrophages, microglial cells and dendritic cells	acquired immunodeficiency syndrome (AIDS)	Vpu co-localizes with the pericentriolar recycling endosome (transferrin and Rab11); Rab11 is required for the Vpu-enhancement of viral particle release	[189]
		Human immunodeficiency virus 1 (HIV-1)	human	CD4+ T cells, macrophages, microglial cells and dendritic cells	acquired immunodeficiency syndrome (AIDS)	The endocytic recycling compartment (transferrin) is used for the transcytosis of HIV-1 in vaginal epithelial cells	[194]
		Human immunodeficiency virus 1 (HIV-1)	human	CD4+ T cells, macrophages, microglial cells and dendritic cells	acquired immunodeficiency syndrome (AIDS)	FIP1C binds to Rab14 and redistributes out of the endosomal recycling complex for Env trafficking and incorporation onto virions towards the surface	[184]
*Flaviviridae*	+ ss RNA	Hepatitis C virus (HCV)	human	hepatocytes (epithelial)	liver damage, cancer or chirrosis	Recycling endosome (Rab11a) is involved in egress of viral cores from the Golgi to cell periphery	[195]
*Herpesviridae*	ds DNA	Herpes simplex virus 1 (HSV-1)	human	epithelial and neuronal cells	forms blisters on or around affected areas - usually the mouth, genitals, or rectum. The blisters break, leaving tender sores	Recycling endosomes (Rab11) are likely involved in internalization of viral glycoproteins from the surface to be included in the capsids	[186]

ds: double-stranded; ss: single-stranded.
